# Cross-species analysis of genetically engineered mouse models of MAPK-driven colorectal cancer identifies hallmarks of the human disease

**DOI:** 10.1242/dmm.013904

**Published:** 2014-04-17

**Authors:** Peter J. Belmont, Eva Budinska, Ping Jiang, Mark J. Sinnamon, Erin Coffee, Jatin Roper, Tao Xie, Paul A. Rejto, Sahra Derkits, Owen J. Sansom, Mauro Delorenzi, Sabine Tejpar, Kenneth E. Hung, Eric S. Martin

**Affiliations:** 1 Oncology Research Unit, Pfizer Global Research and Development, San Diego, CA 92121, USA.; 2 Institute of Biostatistics and Analyses, Faculty of Medicine, Masaryk University, 625 00 Brno, Czech Republic.; 3 Bioinformatics Core Facility, SIB Swiss Institute of Bioinformatics, 1015 Lausanne, Switzerland.; 4 Division of Gastroenterology, Tufts Medical Center, Boston, MA 02111, USA.; 5 The Beatson Institute for Cancer Research, Garscube Estate, Glasgow, G61 1BD, UK.; 6 University Hospital Gasthuisberg, Katholieke Universiteit Leuven, 3000 Leuven, Belgium.; 7 Pfizer Biotherapeutics Clinical Research, Cambridge, 02140 MA, USA.

**Keywords:** *KRAS*, *BRAF*, MAPK, Colorectal cancer, GEMM, Genomic signatures

## Abstract

Effective treatment options for advanced colorectal cancer (CRC) are limited, survival rates are poor and this disease continues to be a leading cause of cancer-related deaths worldwide. Despite being a highly heterogeneous disease, a large subset of individuals with sporadic CRC typically harbor relatively few established ‘driver’ lesions. Here, we describe a collection of genetically engineered mouse models (GEMMs) of sporadic CRC that combine lesions frequently altered in human patients, including well-characterized tumor suppressors and activators of MAPK signaling. Primary tumors from these models were profiled, and individual GEMM tumors segregated into groups based on their genotypes. Unique allelic and genotypic expression signatures were generated from these GEMMs and applied to clinically annotated human CRC patient samples. We provide evidence that a *Kras* signature derived from these GEMMs is capable of distinguishing human tumors harboring *KRAS* mutation, and tracks with poor prognosis in two independent human patient cohorts. Furthermore, the analysis of a panel of human CRC cell lines suggests that high expression of the GEMM *Kras* signature correlates with sensitivity to targeted pathway inhibitors. Together, these findings implicate GEMMs as powerful preclinical tools with the capacity to recapitulate relevant human disease biology, and support the use of genetic signatures generated in these models to facilitate future drug discovery and validation efforts.

## INTRODUCTION

Human sporadic colorectal cancer (CRC) is a complex heterogeneous disease, and this contributes to the low success rate of its clinical trials and lack of robust therapeutics (Betensky et al., 2002; de Bono and Ashworth, 2010). Efforts have been made to understand and account for the heterogeneity of several human cancers, including CRC, with a focus on segmenting cancer populations based on core genetic ‘driver’ lesions (Greenman et al., 2007). In addition, several studies have identified genomic signatures within large CRC datasets that predict clinical outcome (Roth et al., 2010; Dry et al., 2010; Popovici et al., 2012; Budinska et al., 2013; De Sousa E Melo et al., 2013; Sadanandam et al., 2013).

To further understand and experimentally interrogate the biology underlying genetically defined disease segments of interest, and to facilitate discovery of relevant treatment paradigms, stochastic preclinical disease models harboring homologous somatic alterations are crucial. To this end, several studies have utilized genetically engineered model organisms, including *Drosophila* (Vidal and Cagan, 2006; Rudrapatna et al., 2012) and mice (Jonkers and Berns, 2002; Tuveson and Jacks, 2002), to recreate hallmark characteristics of human cancers. *Drosophila* cancer models have shed light on numerous biological underpinnings of cancer, including tumor suppressors, invasion and metastasis (Rudrapatna et al., 2012), providing substrate for further validation in mammalian models. Genetically engineered mouse models (GEMMs) have been utilized as the mammalian cancer model system of choice for decades (Tuveson and Hanahan, 2011; Politi and Pao, 2011). Although GEMMs have traditionally incorporated germline alterations in disease-prevalent genes, models using conditionally controlled, somatically acquired alleles allow a more accurate stochastic modeling of the sporadic nature of human tumorigenesis (Heyer et al., 2010). To address this, GEMMs have been further developed to leverage restricted exposure of Cre recombinase to initiate latent alleles exclusively in tissues of interest, closely mimicking the onset of spontaneous lesions in humans (Johnson et al., 2001; Roper and Hung, 2012; DuPage et al., 2009; Frese and Tuveson, 2007).

To provide maximal experimental utility and enable the translation of preclinical mouse modeling experiments into human disease, GEMMs of human CRC must be driven by homologous allelic series, and exhibit similar clinical presentations to the human disease, including disease histopathology and appearance of metastatic lesions (Heyer et al., 2010; Roper and Hung, 2012). Recently, primary tumors from GEMMs of pancreatic, colorectal and non-small-cell lung cancers harboring genetic lesions that are present in human cancers were shown to be histologically and pathologically similar to their respective human counterparts (DuPage et al., 2009; Hung et al., 2010; Martin et al., 2013). In some cases, GEMMs have closely emulated the response seen in humans to both standard of care and targeted therapies (Arnold et al., 2005); furthermore, the mechanisms of acquired resistance to such agents have often closely resembled those seen in the clinic (Engelman et al., 2008; Jorissen et al., 2009; Van Cutsem et al., 2009; Hegde et al., 2013). Thus, GEMMs are useful preclinical models for modeling human cancer biology and identifying potential therapeutic targets.

**TRANSLATIONAL IMPACT**

**Clinical issue**

Colorectal cancer (CRC) is the third leading cause of cancer mortality in the United States, and ~80% of all cases are sporadic in nature, involving the acquisition of tumorigenic somatic alterations. Treatment options for CRC are limited, and the survival rates associated with advanced-stage disease are low. The highly heterogeneous nature of this disease is thought to contribute to the lack of success of novel therapeutics in the clinic. Thus, preclinical models that recapitulate the core biology of the human disease are needed for the identification of new therapeutic strategies. Despite the heterogeneity associated with sporadic CRC, the vast majority of cases display alterations in a limited number of tumor suppressors and oncogenes. Here, the authors amassed a unique collection of genetically engineered mouse models (GEMMs) harboring conditional alleles that mimic acquired somatic alterations observed in human sporadic CRC, including loss of the tumor suppressors *APC* and *TP53* and gain of oncogenic *BRAF* and *KRAS*. To gain an understanding of the utility of these models, gene signatures were derived and used to stratify genomically heterogeneous clinically annotated patient samples, as well as human cell lines treated with targeted inhibitors.

**Results**

Primary tumors were isolated from GEMMs harboring common CRC ‘driver’ mutations, and these tumors were subjected to gene expression profiling to generate genotype-specific signatures. GEMM-derived signatures were applied to two independent human clinical CRC datasets for which genomic profiling and survival data were available. The GEMM *Kras* signature score was enriched in individuals with a mutation in *KRAS*, and associated with shorter overall survival (OS), relapse-free survival (RFS) and survival after relapse (SAR). Interestingly, the signature further segregated the *KRAS* mutant CRC patient population into two clinically distinct groups, consistent with emerging evidence of heterogeneity in this population in both gene expression and survival. Finally, the signature was predictive of response to MEK inhibitors, which are widely used as cancer drugs, in human CRC cell lines.

**Implications and future directions**

Together, these results demonstrate that gene signatures derived from genetically and contextually relevant GEMMs are capable of further resolving genomically heterogeneous populations of human CRC and identifying patients with characteristics of aggressive disease. The correlation of the GEMM *Kras* signature with response to targeted inhibition of a clinically relevant pathway in a collection of human CRC cell lines highlights its potential utility in predicting therapeutic response. Future studies will focus on the application of this signature to other therapeutic modalities of interest, and on further understanding the contribution of key nodes or targets present within the signature itself. On a wider scale, this study demonstrates the usefulness of GEMMs expressing conditional alleles for exploring genetic heterogeneity in human malignancies.

To further our understanding of the molecular etiology underlying common genotypic subsets of human CRC, and to assess the extent to which they recapitulate human disease in animal models, we amassed a collection of GEMMs that combine colon-specific mutations, including somatic alterations in *Apc* (*Apc*^CKO^), *Tp53* (*Tp53*^flox/flox^), *Kras* (*Kras*^LSL-G12D^) and *Braf* (*Braf*^V600E^), genes that are among the most frequently mutated in human sporadic CRC (Cancer Genome Atlas Network, 2012). Primary tumor material from this collection was subjected to gene expression profiling to assess core similarities and differences among these models, and to generate unique signatures based on genotype. These signatures were then evaluated in human CRC tissue with annotated clinical data to assess the ability of these GEMMs to recapitulate the core transcriptional biology of their human CRC counterparts. Overlapping gene expression modules shared between GEMM and human signatures represent potential points of therapeutic interrogation and provide key substrate for follow-up validation and drug discovery efforts.

## RESULTS

### Development and profiling of genetically relevant CRC GEMMs

**Fig. 1.**
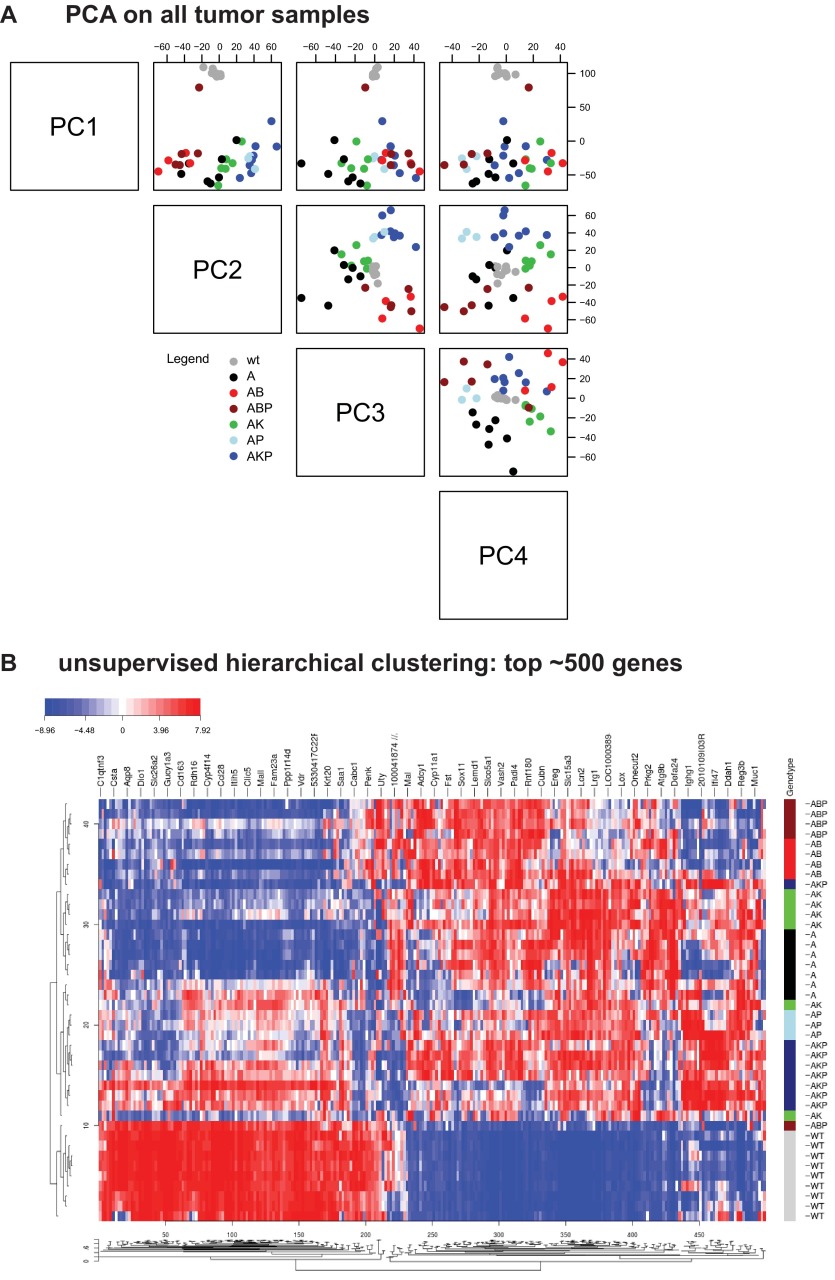
**GEMM primary tumors segregate by genotype.** (A) Principal component analysis (PCA) on GEMM primary tumor samples and normal colon tissue. wt, normal colon from wild-type untreated mice. The following designations describe the alleles present in CRC GEMMs: A, *Apc*; AB, *Apc*, *Braf*; ABP, *Apc*, *Braf*, *Tp53*; AK, *Apc*, *Kras*; AKP, *Apc*, *Kras*, *Tp53*; AP, *Apc*, *Tp53*. (B) Unsupervised hierarchical clustering using the top 500 most differentially expressed genes from samples as described in A. WT, wild type.

Adult GEMMs harboring combinations of latent, inactive alleles of the four most common somatic lesions observed in human CRC (Cancer Genome Atlas Network, 2012) (*APC*, *TP53*, *KRAS* and *BRAF*) were subjected to surgically restricted delivery of *AdCre* to the distal colon; mice were then followed longitudinally for tumor progression via endoscopy, and tumor material was harvested as previously described (Hung et al., 2010; Martin et al., 2013). The conditional *Apc* and *Tp53* alleles harbor *loxP* sites (floxed), which, upon exposure to *AdCre*, result in excision of critical exons, resulting in loss-of-function proteins, as previously described (Kuraguchi et al., 2006; Kirsch et al., 2007). The conditional *Kras* and *Braf* alleles harbor floxed transcriptional stop elements upstream of mutant forms of exon 1 (*Kras*^G12D^) (Hung et al., 2010) or exon 15 (*Braf*^V600E^) (Coffee et al., 2013). A list of primary tumors with allelic combinations is provided (supplementary material Table S1). Tumors and normal colonic tissue from wild-type littermate controls were subjected to whole-genome expression profiling. Subsequently, principal component analysis (PCA) and unsupervised hierarchical clustering on the top 500 most variable genes was performed. Individual CRC GEMMs clustered by genotype, both in the PCA (Fig. 1A, genotype representing the first two principal components) and hierarchical clustering (Fig. 1B). These results demonstrate that the genotypes of these models represent the primary differentiating feature, and suggest that each genotype likely possesses unique underlying biological characteristics.

### Allele-specific GEMM signatures

**Fig. 2.**
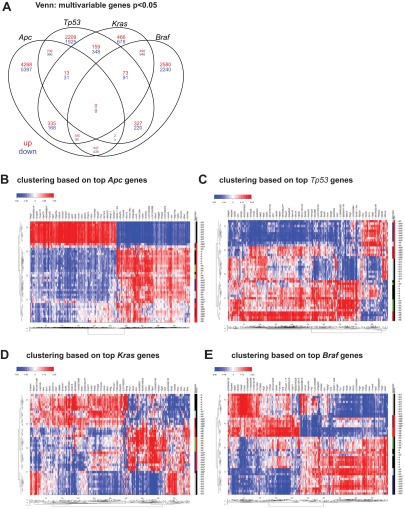
**Multivariable analysis identifies genes associated with each allele from the GEMM cohort.** (A) Venn diagram depicting the number of unique or shared genes associated with each GEMM allele. Red, upregulated genes; blue, downregulated genes. (B–E) Clustering of GEMMs based on expression profiles of genes associated with each allele.

To further assess the underlying differences among our CRC models, we identified gene signatures (lists of differentially expressed genes) characteristic of each mutant allele (*Apc*, *Tp53*, *Kras*, *Braf*) within the GEMM collection using a multivariable analysis (see Materials and Methods). It is important to note that all GEMMs contain *Apc* lesions; therefore, all results for *Braf*, *Kras* and *Tp53* alleles should be interpreted with this regard. A Venn diagram (Fig. 2A) and heatmaps of supervised hierarchical clustering on the signature-specific genes (Fig. 2B–E) demonstrate that these gene lists partially overlap, suggesting common biological characteristics, including redundant signaling and pathway activation. To determine whether the unique or intersecting gene lists associated with each mutant allele displayed enrichment in known biological processes or curated gene signatures, we cross-referenced each to the molecular signatures database [MSigDB (www.broadinstitute.org/gsea/msigdb/)]. Indeed, common gene sets enriched among upregulated *Kras* and *Braf* genes included several annotated MAPK pathway sets, consistent with the established roles of mutant *Kras* and *Braf* in activating this pathway (supplementary material Table S2). Gene sets enriched among shared upregulated *Apc* and *Tp53* genes included several cell cycle gene sets as well as DNA synthesis, replication and repair, consistent with their established roles as tumor suppressors and thus with the deregulation of these functions in our models (supplementary material Table S3). Gene sets enriched among unique genes for each allele were also assessed. Gene sets found to be enriched in *Kras*-specific genes included metabolism, signaling downstream of receptors, and adhesion (supplementary material Table S4), functions previously ascribed to mutant *KRAS* (Racker et al., 1985; Pollock et al., 2005; Rajalingam et al., 2007; Levine and Puzio-Kuter, 2010). Interestingly, gene sets enriched among unique *Braf* genes also include metabolism, consistent with previously established links between oncogenic *BRAF* and metabolic deregulation (Yun et al., 2009); however, additional gene sets included immune response signaling, consistent with additional roles for oncogenic *BRAF* (Sumimoto et al., 2006) (supplementary material Table S5). Gene sets found to be enriched in *Apc*-specific genes included development (supplementary material Table S6), consistent with the role of aberrant *APC* in WNT–β-catenin signaling and development (Clevers, 2006), as well as several gene sets associated with small-molecule transport, a role to our knowledge not fully characterized for aberrant *APC*. Gene sets enriched in *Tp53*-specific genes included ubiquitylation and proteolysis pathways (supplementary material Table S7), consistent with the central role of these pathways in regulating endogenous *TP53* (Lee and Gu, 2010). Taken together, these findings indicate that lesions in our GEMM alleles of interest result in gene signatures characteristic of known or putative biological roles for each allele.

### Generation and validation of GEMM allelic signatures

**Fig. 3.**
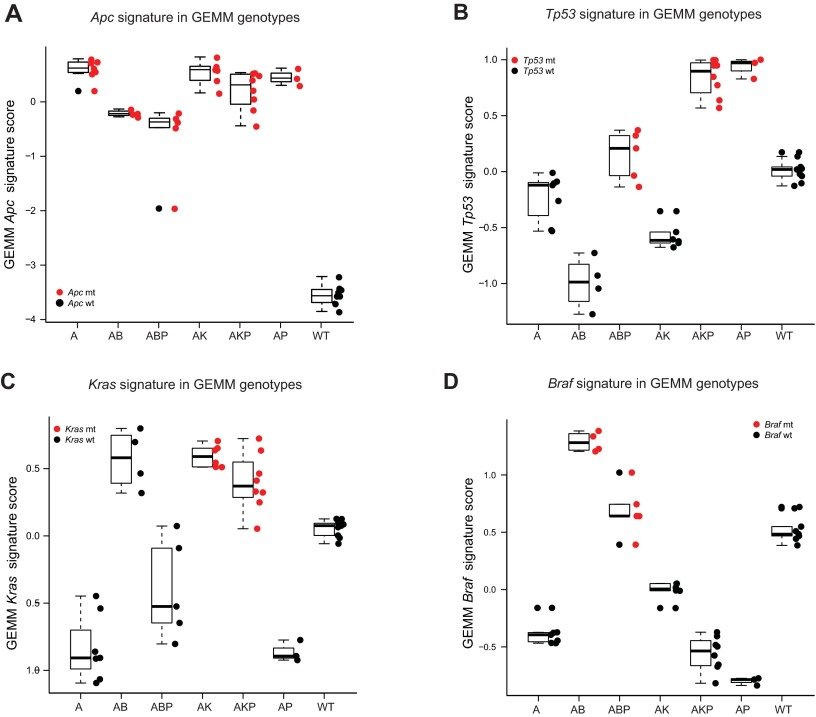
**Internal validation of the GEMM signatures to distinguish models containing mutant alleles.** (A–D) GEMM allele-specific signatures were generated (*Apc*, *Tp53*, *Kras*, *Braf*), and a signature score was calculated in each of the six GEMM genotypic models as well as WT normal colon control.

We defined GEMM allele-specific scores as a difference of average gene expression between the top 100 up- and top 100 downregulated genes from the corresponding signature. The score for each individual GEMM allelic signature (*Kras*, *Braf*, *Apc*, *Tp53*; supplementary material Tables S8–S11, respectively) was computed in each of the models (A: *Apc*; AK: *Apc*, *Kras*; AKP: *Apc*, *Kras*, *Tp53*; AB: *Apc*, *Braf*; ABP: *Apc*, *Braf*, *Tp53*; AP: *Apc*, *Tp53*; WT, wild type; supplementary material Table S1). As expected, the models containing a given mutation had the highest score for that allelic signature in the discovery set (Fig. 3A–D). For instance, the GEMM *Apc* signature score was high in all GEMM models, because all models contain this mutation (Fig. 3A), whereas the GEMM *Tp53* signature was high in models containing *Tp53*, including AP, ABP and AKP, but low in A, AB and AK (Fig. 3B). In the case of the GEMM *Kras* signature, the score was high in models containing *Kras*, including AK and AKP (Fig. 3C). The highest *Braf* score was found in models containing *Braf*, including AB and ABP (Fig. 3D). Interestingly, the GEMM *Kras* score was also high in models with *Braf* and *Apc* mutation (AB), but not in those containing *Braf*, *Apc* and *Tp53* mutation (ABP) (Fig. 3C), suggesting that the addition of *Tp53* to the *Apc*, *Braf* mutant background might result in less reliance on MAPK-driven signaling. Similar trends were seen in other genotypes, with *Tp53* mutation leading to a systematically lower signature score compared with their counterparts without the mutation (*Apc* signature in AP versus A, Fig. 3A; *Kras* signature in AKP versus AK, Fig. 3C; ABP versus AB, Fig. 3D). A potential explanation for these observations could include the increased presence of genomic instability, a well-known consequence of aberrant *Tp53*.

We next applied the signature to an independent GEMM CRC sample set consisting of acute activation of shared alleles, including *Apc*, *Tp53* and *Kras*. Consistent with the findings in our discovery cohort, our GEMM allelic signatures scored highest in GEMMs derived from an independent cohort that contained the corresponding mutant allele (supplementary material Fig. S1A–C), further validating their predictive utility.

### Overlap of allele-specific GEMM *Kras* and *Braf* signatures with clinically annotated CRC datasets

**Fig. 4.**
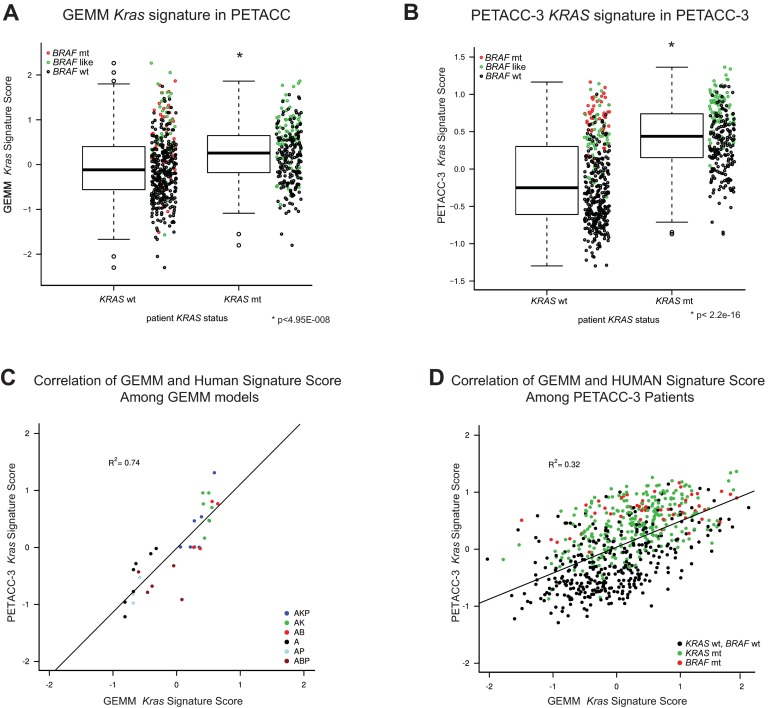
**GEMM *Kras* signature can distinguish *KRAS* mutant patients.** (A) Box plot depicting distribution of IQR normalized GEMM *Kras* signature score in PETACC-3 patients that are *KRAS* wild-type (wt) or mutant (mt). *KRAS* wild-type patients were further annotated as *BRAF* mutant (red) or containing significant enrichment of a *BRAF*-like signature (Popovici et al., 2012) (green). (B) Box plot depicting distribution of IQR normalized PETACC-3 *KRAS* signature score in PETACC-3 patients. *KRAS* wild-type patients were further annotated as in A. (C,D) Correlation between IQR normalized *KRAS* scores derived from PETACC-3 (*x*-axis) or GEMM (*y*-axis) signatures among the GEMM collection (C) and PETACC-3 patients (D). Solid line represents linear fit. Colors in C represent each GEMM genotype. Colors in D represent status of *KRAS* and *BRAF* mutation in PETACC-3 patients: red, *BRAF* mutant; green, *KRAS* mutant.

To assess the extent to which our GEMMs recapitulate the genetic and biological features of human CRC, and to assess the utility of this collection for preclinical studies, we compared their genomic signatures to those of clinically annotated human CRC datasets. To this end, we utilized the Pan-European Trials in Alimentary Tract Colon Cancers (PETACC-3), a large Phase III randomized trial in which 688 patients with stage II or III CRC were characterized by genomic and mutational analysis, including *KRAS* and *BRAF*. Because the mutant *Kras* allele in the GEMM cohort (*Kras*^LSL-G12D^) is a gain-of-function mutation, for the purpose of comparison we considered all *KRAS* gain-of-function mutations in the PETACC-3 dataset, with the caveat that different types of *KRAS* mutations potentially have unique biological characteristics (Kirk, 2011). As indicated in Fig. 4A, the average GEMM *Kras* signature score was significantly higher in patients with the *KRAS* mutant than those with wild-type *KRAS*. Given the variability in the GEMM *Kras* signature score among individuals with wild-type *KRAS* and the fact that our *Kras* signature scored high in our *Braf*-containing models, possibly picking up on common MAPK pathway mechanisms, these patients were further annotated based on *BRAF* mutation or similarity to a published *BRAF*-like signature (Popovici et al., 2012). Interestingly, of the *KRAS* wild-type patients, both *BRAF* mutant (Fig. 4A, red circles) as well as those with a high *BRAF*-like signature score (Fig. 4A, green circles) tended to display a higher signature score, supporting our hypothesis that, in addition to distinguishing *KRAS* mutant patients, the GEMM *Kras* signature also captures those with high MAPK pathway activity. Together, these data indicate that the GEMM signature is enriched in patients with *KRAS* mutation, as well as *BRAF* mutation or a high degree of similarity to a *BRAF*-like signature, the latter of which is potentially indicative of a common biology shared among *KRAS* and *BRAF* mutant patients.

To determine whether our GEMM *Kras* signature is representative of human *KRAS* mutant CRC tumors, we compared it to a human *KRAS* signature derived in the multivariable model with *KRAS* and *BRAF* mutation as covariates in PETACC-3 patients. Consistent with the GEMM, the PETACC-3 *KRAS* signature score was higher among *KRAS* mutant patients than *KRAS* wild-type patients, whereas, again, *BRAF* mutant and *BRAF*-like patients tended to score highest among the *KRAS* wild-type population (Fig. 4B). The GEMM and PETACC-3 *KRAS* signature scores showed a high degree of correlation both among GEMMs (Fig. 4C, *R*^2^=0.74) and among patients (Fig. 4D, *R*^2^=0.32). These findings suggest that the *Kras* signature derived from a relatively homogeneous background such as the GEMM might be capable of capturing common and disease-relevant biology present in human *KRAS* patients.

Interestingly, our GEMM *Braf* signature score did not correlate with the human *BRAF* signature score of Popovici et al. (Popovici et al., 2012), nor was it able to predict *BRAF* mutant tumors in the PETACC-3 data. Also, the recent *BRAF* signature derived from human samples did not predict correctly *Braf* mutant status in our GEMMs (data not shown). This, together with the results of the *Braf* signature pathway analysis pointing to proliferation, shows that our *Apc*-based *Braf* models are potentially less representative of the human *BRAF* mutant population. This is consistent with the low frequency of concomitant *BRAF* and *APC* lesions observed in human cases (Cancer Genome Atlas Network, 2012).

### Clinical characteristics of patient samples based on GEMM *Kras* signature score

We assessed differences in available clinical variables among all individuals in the PETACC-3 cohort. Patient populations were defined based on each GEMM signature score into allele-like and non-allele-like groups (threshold 0 on inter-quartile range normalized scores). GEMM *Kras*-like tumors exhibited a statistically significant enrichment for various characteristics, including mucinous histology, *KRAS* mutant, *BRAF* mutant, right-side, stage 3, and similarity to a *BRAF*-like population shown previously to be associated with poor prognosis (Popovici et al., 2012) (supplementary material Table S12), implicating the ability of the GEMM *Kras* signature at distinguishing aspects of advanced disease.

### GEMM *Kras* signature is associated with poor outcome

**Fig. 5.**
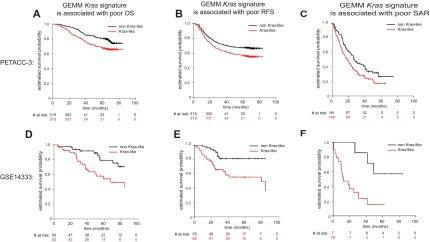
**GEMM *Kras* signature is predictive of poor prognosis in two independent clinically annotated CRC patient datasets.** (A–C) Kaplan-Meier estimates of three survival end points among PETACC-3 patients (OS, RFS and SAR) between *Kras*-like (red) and non *Kras*-like (black) groups, as defined according to the *Kras* gene expression signature derived from GEMMs. (D–F) Same as A–C but for patients within the GSE14333 dataset. Survival times were cut at 84 months.

To determine whether the GEMMs are representative of advanced disease, we examined survival differences among annotated patients in PETACC-3. Differences in overall survival (OS), relapse-free survival (RFS) and survival after relapse (SAR) were compared. To validate our findings, we performed a similar assessment on an independent publicly available sample cohort (GEO GSE14333) (Jorissen et al., 2009), consisting of 115 stage II/III human CRC samples with gene expression profiling and survival data. Of the four core GEMM signatures generated (*Apc*, *Tp53*, *Braf*, *Kras*), the *Kras* signature score produced the highest hazard ratios for OS and SAR in the PETACC-3 dataset, and among the highest hazard ratios for OS, RFS and SAR in the GSE14333 dataset (Table 1), suggesting that it is most indicative of advanced disease. OS, RFS and SAR based on GEMM *Kras* signature was plotted for the PETACC-3 dataset (Fig. 5A–C) and for the GSE1433 dataset (Fig. 5D–F). Additional Kaplan-Meier plots for GEMM *Braf*, *Apc* and *Tp53* signatures in PETACC-3 as well as GSE144333 can be found in supplementary material Figs S2 and S3, respectively. Because the GEMM *Kras* signature was associated with some prognostic clinical variables (e.g. stage), we also fitted a multivariable survival model with GEMM *Kras*-like signature, *BRAF* mutant, *KRAS* mutant, mucinous status, grade and MSI, within stage-3 patients of the PETACC-3 dataset (stage 2 patients were enriched for relapsed patients, so were not representative of the population). The GEMM *Kras* signature remained significant for both OS and RFS (supplementary material Table S13). Together, these findings suggest that our GEMM *Kras* signature could offer insight into survival characteristics in two independent large human CRC patient cohorts.

**Table 1.**
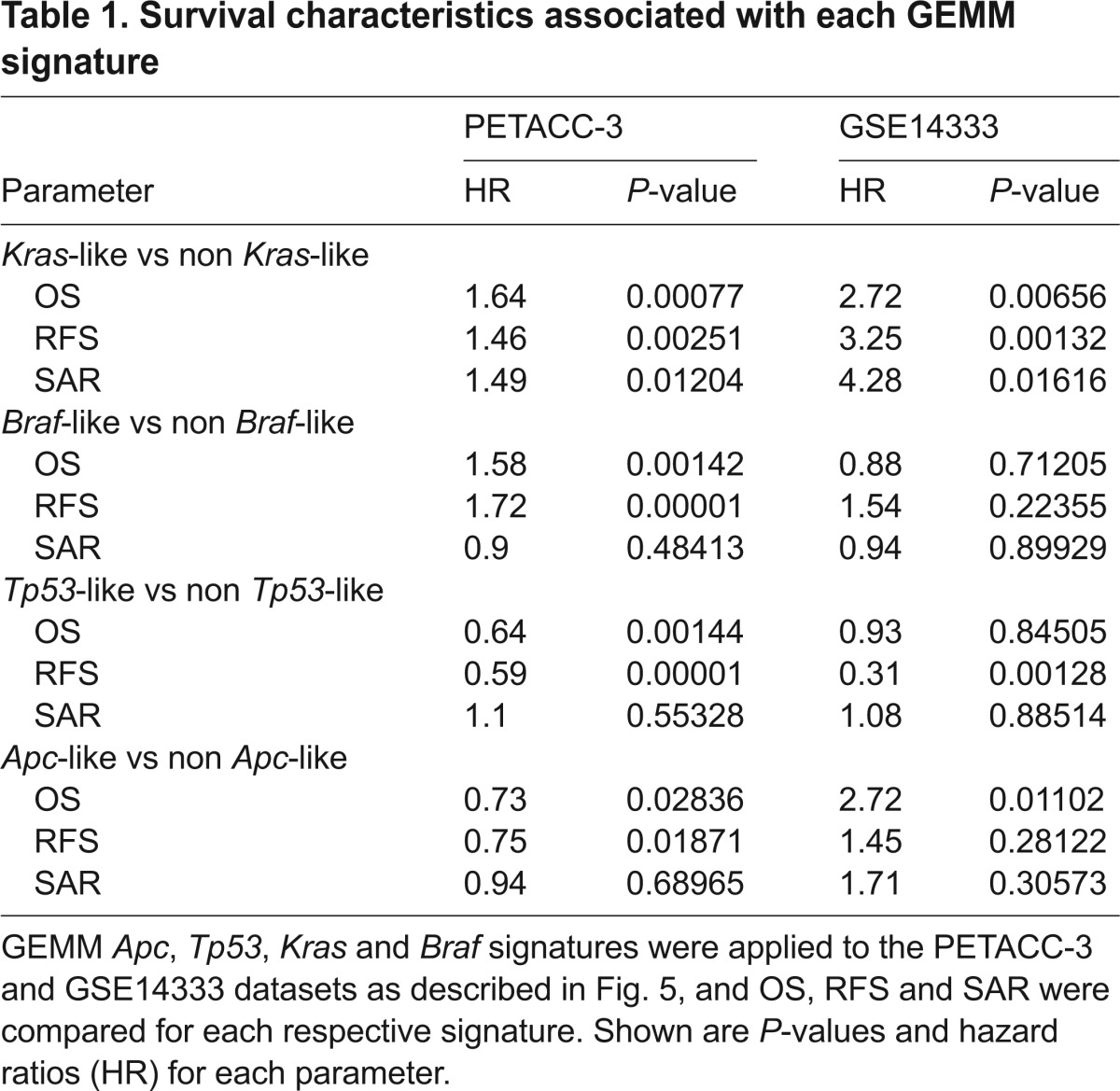
Survival characteristics associated with each GEMM signature

**Fig. 6.**
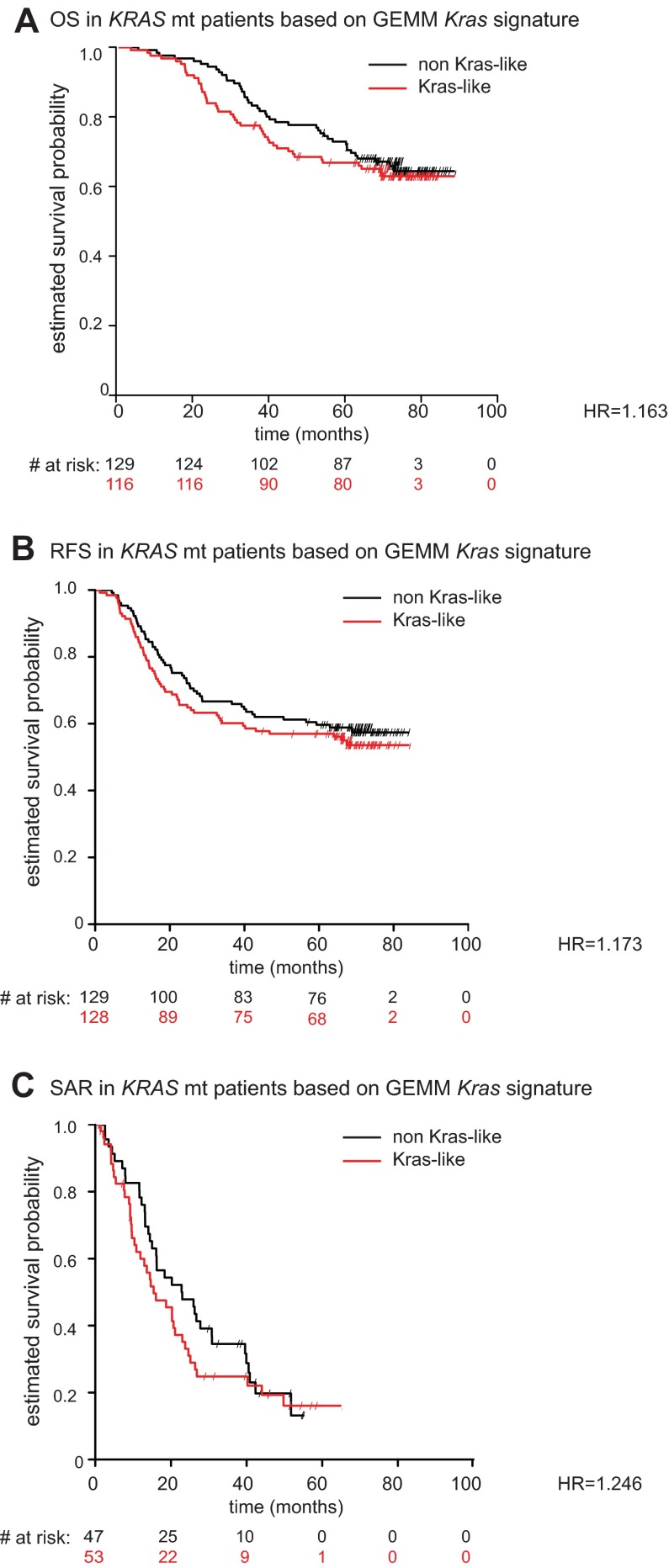
**GEMM *Kras* similarity score assigned within the PETACC-3 *KRAS* mutant population is associated with poor prognosis.** (A–C) The PETACC-3 *KRAS* mutant population was treated separately and a similarity score was assigned to each *KRAS* mutant patient, based on similarity to the GEMM *Kras* signature. Kaplan-Meier curves demonstrating that the GEMM *Kras* signature is predictive of poor overall survival (OS, A), relapse-free survival (RFS, B), and survival after relapse (SAR, C) within the PETACC-3 *KRAS* mutant population. Survival times were cut at 84 months.

Given that *KRAS* mutant CRC patients have been shown to be heterogeneous (Budinska et al., 2013; Sadanandam et al., 2013) and given the ability of the GEMM *Kras* signature to distinguish patients with poor prognosis, we sought to determine whether this signature could further delineate clinical features, specifically in a *KRAS* mutant patient population. Although not statistically significant, a trend toward worse prognosis was observed for *KRAS* mutant patients with high GEMM *Kras* signature score for OS, RFS and SAR (Fig. 6A–C, *P*=0.480, *P*=0.398 and *P*=0.341, respectively).

Together, these data indicate that the GEMM *Kras* signature can distinguish a subpopulation of patients with poor prognosis, perhaps owing to its ability to further distill a heterogeneous patient population to the core underlying biology beyond simply the status of a given driver lesion, much like the recent *BRAF* signature (Popovici et al., 2012) with which it is correlated.

### GEMM *Kras* signature is predictive of sensitivity to targeted inhibitors

**Fig. 7.**
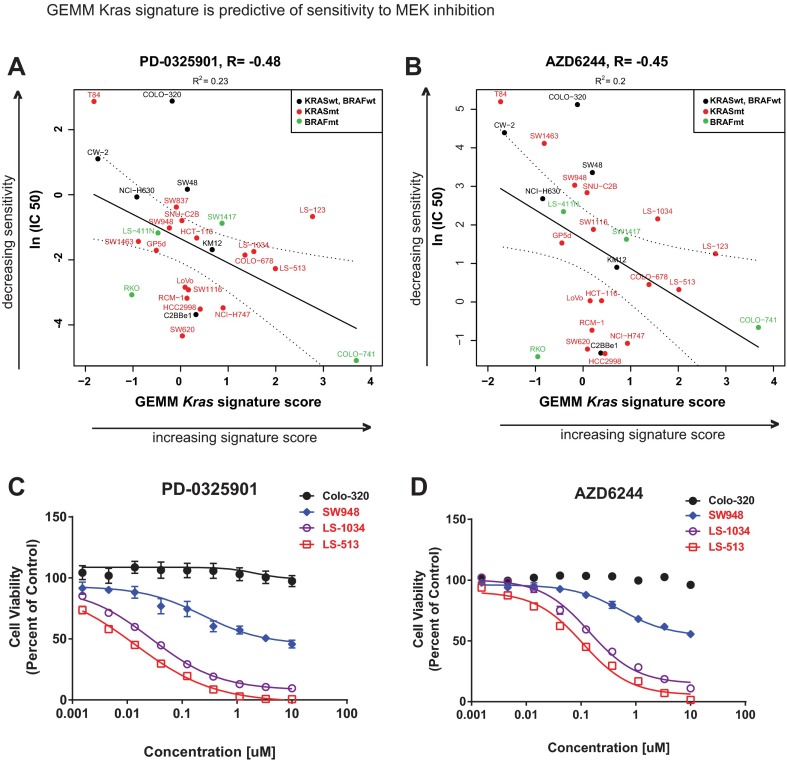
**GEMM *Kras* predicts sensitivity to targeted MEK inhibition.** The top 100 most significant GEMM *Kras* signature genes were used to segregate cell lines based on similarity to these genes (*x*-axis, GEMM *Kras* signature score; increasing value indicates increasing signature score), and compare this to the relative sensitivity to targeted MEK inhibitors reported in the Sanger dataset (www.cancerrxgene.org) [*y*-axis, ln (IC_50_); increasing value indicates decreasing sensitivity to the inhibitor], including PD-0325901 (A) and AZD6244 (B). *KRAS* mutant cell lines are in red, *BRAF* mutant cell lines are in green, *KRAS/BRAF* wild-type cell lines are in black. The associated Pearson correlations and *R*^2^ values relating *Kras* signature score to inhibitor sensitivity are shown above each graph. (C,D) Independent confirmation of sensitivity to MEK inhibitors. Representative cell lines with relatively high (LS-1034, LS-513) and low (Colo-320, SW948) GEMM *Kras* signature scores were experimentally tested as an independent assessment of sensitivity to MEK inhibitors PD-0325901 (C) and AZD6244 (D). *KRAS* mutant cell line names are in red, *KRAS/BRAF* wild-type cell line names are in black.

To determine the utility of the GEMM *Kras* signature as a preclinical model selection tool, we assessed its ability to predict response to targeted inhibitors in a panel of cell lines. Given the clinical potential in applying MEK inhibitors to treat various tumor types, including CRC, we sought to determine whether the GEMM signature was predictive of response to these inhibitors as determined by a publicly available study of drug sensitivity across a comprehensive collection of cancer cell lines (http://www.cancerrxgene.org), with a focus on CRC. A high GEMM *Kras* signature score was associated with increased sensitivity of CRC cell lines to two independent MEK inhibitors used in the study, PD-0325901 and AZD6244 (Fig. 7A,B, respectively). To independently validate these findings, we selected representative cell lines with relatively high and low GEMM *Kras* signature scores (high: LS-1034, LS-513; low: Colo-320, SW948), and assessed cell viability following a full-dose response of these MEK inhibitors. The cell lines with higher GEMM *Kras* signatures displayed relatively greater sensitivity than those lines with lower GEMM *Kras* signatures to the MEK inhibitors PD-0325901 and AZD6244 (Fig. 7C,D, respectively). This supports our hypothesis that the GEMM *Kras* signature is associated with an increased dependency on MAPK signaling, and therefore an enhanced sensitivity to pathway inhibition via selective targeting of MEK. This is consistent with the known ‘driver’ phenotype of mutant *KRAS* and the increased dependency on the MAPK pathway observed in several *KRAS* mutant cell lines. Interestingly, the GEMM *Kras* signature score added predictive utility beyond simply *KRAS* mutation status of the cell lines: a signature score positively correlated with sensitivity to MEK inhibition, even within a set of *KRAS* mutant cell lines. Taken together, these findings provide motivation for using the GEMM *Kras* signature for predicting response to targeted inhibitors of the MAPK pathway, including those targeting MEK.

## DISCUSSION

The identification of core ‘driver’ lesions among tumor indications provides a means for segmenting patients and, in some cases, selecting treatment regimens. Despite advances in patient stratification and treatment selection, there are still sizeable segments of human disease with limited effective treatment options. One such segment is defined by the presence of *KRAS* mutations, constituting roughly 30–40% of sporadic CRC (Jorissen et al., 2009; Cancer Genome Atlas Network, 2012). Further compounding this problem is the lack of informative preclinical models in which to conduct rapid drug discovery efforts.

Next-generation GEMMs have gained prominence as preclinical cancer models (DuPage et al., 2009; Heyer et al., 2010; Politi and Pao, 2011). Specific advantages of these models include the ability to selectively activate latent alleles of interest, effectively modeling the stochastic gain of activating mutations and/or loss of tumor suppressors commonly observed in sporadic human cancers. Our GEMM collection contains combinations of genes frequently mutated or lost in human CRC, including *Apc*, *Tp53*, *Braf* and *Kras*, thereby allowing us to model a broad spectrum of human disease. Adding to the utility of these models, primary tumors are used as substrate to generate tumor-derived cell lines that maintain much of the biology of the original tumors, and retain key alleles of interest (Martin et al., 2013). Further, these cell lines serve as a platform for *in vitro* and *in vivo* interrogation because they are amenable to growth in subcutaneous space, in sites common for metastasis such as the liver, and in the native colonic environment of syngeneic, immunocompetent recipients (Martin et al., 2013). As in any GEMM, there are also clear drawbacks to these models, such as the limited number of defined genetic lesions and tumor heterogeneity relative to their human counterparts, in large part due to the inherent nature of an inbred model. In addition, owing to their historically short lifetime as preclinical models, their translational value of has yet to be fully realized. Thus, it is important to understand the role of these models as a complementary tool in a larger comprehensive preclinical drug discovery program.

In the current study, we investigated the genomic characteristics of primary tumors from our collection of CRC GEMMs containing genetic lesions that are present in a large portion of human disease cases. The genomic profiles of these tumors properly segregated based on their core genotypes, with each genotype containing unique distinguishing signatures. Our *Braf* models were exclusively generated along with loss of *Apc*, a condition likely not indicative of human CRC progression as indicated by a recent assessment of human CRC mutational data (Cancer Genome Atlas Network, 2012) and also reflected in our GEMM *Braf* signature failing to classify *BRAF* mutant clinical samples.

The GEMM *Kras* signature was effectively validated within an independent collection of GEMMs, as it properly distinguished *Kras* mutant models from non-mutant. A more detailed analysis of the GEMM *Kras* signature revealed that it was enriched in human CRC patients with advanced disease and poor prognosis. The signature was also able to further stratify the *KRAS* mutant segment of a large clinical cohort, suggesting that a comprehensive signature can provide additional power in further segregating a patient population of interest, beyond simply the status of a given driver lesion, and indicating that there are likely additional underlying characteristics that account for severity of disease beyond the mutation status of *KRAS*. Finally, the signature provided additional utility in predicting sensitivity to targeted MEK inhibition across a panel of CRC cell lines, because those lines with a high signature score tended to display increased sensitivity to two independent MEK inhibitors, suggesting a utility in predicting pathway dependence. The correlation was maintained even within a set of cell lines that harbor *KRAS* mutation: *KRAS* mutant cell lines with relatively higher signature scores displayed increased sensitivity compared with mutant lines with lower signature scores. This approach could potentially be used to identify additional pathway dependencies and corresponding therapeutic sensitivities. Taken together, this study highlights instances in which signatures generated from the GEMMs are applicable to recapitulating biological characteristics of human disease, including prognosis and response to targeted therapeutics. Although several limitations preclude the use of GEMMs as a stand-alone discovery model, the features described herein provide further insight into the power of these GEMMs of sporadic CRC as a companion preclinical discovery model in a comprehensive drug discovery effort.

## MATERIALS AND METHODS

This research protocol was approved by our attending veterinarian, and by the Pfizer Institutional Animal Care and Use Committee (IACUC).

### CRC GEMMs

The generation and genotyping of *Apc* (*Apc*^CKO^), *Tp53* (*Tp53*^flox/flox^), *Kras* (*Kras*^LSL-G12D^) and *Braf* (*Braf*^V600E^) genetically engineered mice has been previously described (Hung et al., 2010).

### CRC GEMM tumor samples and gene expression data

Murine primary tumor samples from GEMMs treated with AdCre, and normal colon tissue from untreated wild-type mice were collected. Wild-type mouse colon tissue used for RNA extraction and microarray analysis was enriched for epithelial cells. Briefly, colons were opened lengthwise, cut into 3–5 mm fragments, and washed in HBSS-glucose. Fragments were then resuspended in 20 ml HBSS-glucose-dispase-collagenase solution, placed into a conical tube and agitated on a shaking platform for 25 minutes at 25°C. The digested tissue was further disaggregated by hand pipetting and vigorous shaking for 3 minutes and inspected under an inverted microscope. Subsequently, enzymes were neutralized with 50 ml DMEM-sorbitol and crypt cell suspensions were separated from intestinal fragments and passed through a 70-μm cell strainer. The epithelial-enriched fraction was briefly centrifuged and used for RNA extraction and microarray analysis. RNA was isolated and processed for hybridization on Mouse Affymetrix GeneChip 430 2.0 arrays (Affymetrix, Santa Clara, CA) as previously described (Martin et al., 2013). All gene expression data can be found at the Gene Expression Omnibus (www.ncbi.nlm.nih.gov/geo/) under accession number GSE50794. Our training set consisted of Affymetrix Mouse 430 2.0 gene expression profiles of 33 primary tumors representing the following genotypes: *Apc* (7), *Apc/Kras* (6), *Apc/Kras/Tp53* (8), *Apc/Tp53* (3), *Apc/Braf* (4), *Apc/Braf/Tp53* (5) and nine normal colon tissue samples.

The validation set consisted of Affymetrix Mouse 430 2.0 gene expression profiles of 15 primary tumors of genotypes: *Apc* (3), *Apc/Kras* (6), *Apc/Tp53*(6) and three normal colon tissues.

### Clinical and cell line data

803 stage II or III human CRC gene expression profiles from both the PETACC-3 trial [688 formalin-fixed paraffin-embedded samples profiled on ALMAC CRC DSA platform (Almac, Craigavon, UK) (Budinska et al., 2013)] and Moffit samples [115 fresh frozen samples profiled on Affymetrix HG U133+ 2.0 platform (Jorissen et al., 2009)] with available clinical and survival data were used to test whether our GEMM models are representative of human disease. The PETACC-3 data are available from the Array Express database under the accession number E-MTAB-990; the Moffit data are available from the GEO database under accession number GSE14333. Cell line gene expression profiles with drug sensitivity (http://www.cancerrxgene.org) (Garnett et al., 2012) profiled on Affymetrix HG U133A platform (Affymetrix, Santa Clara, CA) were downloaded from the Array Express database under the accession number E-MTAB-783.

### Microarray data normalization and data filtering

All Affymetrix gene expression data were normalized and summarized using the function three step of affyPLM R package (www.bioconductor.org) with default settings, background correction, quantile normalization and median polish probe summarization. ALMAC gene expression profiles from the PETACC-3 trial were processed as previously described (Popovici et al., 2011; Popovici et al., 2012). In each dataset, one probeset with the highest variability was selected as a representative of each EntrezGene ID. The variability for each probeset was estimated by robust linear regression (rlm function in R package MASS) as the robust scale estimate (RSE). This resulted in the following number of EntrezGene IDs: 21,758 in GEMM datasets, 14,926 in PETACC-3 dataset, 20,752 in GSE 14333 dataset and 11,237 in the cell line dataset. For all analyses with clinical data, an overlapping set of 13,265 EntrezGene IDs between the two clinical datasets (from ALMAC and Affymetrix platforms) was used. For signature development, mouse EntrezGene IDs were matched to their human homologs, reducing the number of EntrezGene IDs to 15,888 and intersected with 13,265 EntrezGene IDs of clinical datasets, leading to a final subset of 11,745 EntrezGene IDs.

### Statistical analysis, clustering and classifier development

A multivariable linear additive model was built on a GEMM training set of 15,888 EntrezGene IDs to estimate mutation-allele-specific (*Apc*, *Kras*, *Braf*, *Tp53*) effects, with WT in all alleles as baseline. The genes that were assigned a statistically significant effect in a given mutation made up the mutation-specific gene list. Unsupervised hierarchical clustering with average linkage and Pearson correlation as a measure of similarity was used to cluster sets of the top 500 most variable EntrezGene IDs and then the top 500 most variable allele-specific genes and samples. For classifier construction, the final subset of 11,745 human homolog EntrezGene IDs was used.

The top 100 up- and downregulated genes from multivariable analysis specific for a given allele were used to define the allele-specific score, defined as a difference of average gene expression between up- and downregulated genes of the allele. The rule score >0 served as classifier defining allele-like group, except for the *KRAS* mutant subpopulation, where the median of the *KRAS*-like score was taken as threshold. Prior to application of the classifier and consequent survival analysis, the genes in the datasets were median-centered and normalized by inter-quartile range.

### MSigDB analysis

Gene lists associated with each mutant allele (*Kras*, *Braf*, *Apc*, *Tp53*) generated from the multivariable analysis above (*P*<0.01 regulated for each allele) were uploaded to the MSigDB analysis tool [Broad Institute (http://www.broadinstitute.org/gsea/msigdb/index.jsp)]. Enrichment in MSigDB gene sets from all major canonical pathway collections were assessed and ranked by *P*-value. The top 10–20 MSigDB gene sets with the most significant enrichment for each allelic gene list were identified.

### Comparison of GEMM *Kras* signature score and cell line sensitivity

GEMM *Kras* signature score classifier was applied to normalized, EntrezGene ID summarized cell line dataset (http://www.cancerrxgene.org). For this purpose, 66 upregulated and 74 downregulated EntrezGene IDs from the original GEMM *Kras* classifier that were found on the Affymetrix HG U133A platform were used to calculate the GEMM *Kras* score for each CRC cell line in this dataset. This score was then plotted with the corresponding IC_50_ values of drug response to the MEK inhibitors PD-0325901 and AZD6244 for each cell line, as reported in this dataset, and a linear model was fitted.

### Independent confirmation of cell line sensitivity to MEK inhibitors

An independent validation of sensitivity to MEK inhibitors PD-0325901 and AZD6244 based on GEMM *Kras* signature score was performed by selecting representative cell lines with relatively high GEMM *Kras* signature scores (LS-1034, LS-513) and low signature scores (Colo320, SW948). Briefly, cell lines were seeded at 1000 cells/well in 96-well culture plates in growth medium with 10% FBS. Cells were incubated overnight and treated with DMSO (0.1% final) or serial diluted compound for 4 days. Cell viability was assessed adding Cell Titer Glo reagent (CTG, Promega, Madison, WI) and plates were incubated at room temperature for 30 minutes. Luminescence signals were read and IC_50_ values were calculated by plotting luminescence intensity to drug concentration in nonlinear curves using GraphPad Prism (GraphPad, La Jolla, CA).

### Survival analysis

Outcome variables were overall survival (OS), relapse-free survival (RFS) and survival after relapse (SAR). Survival probabilities were estimated using the Kaplan-Meier method, and Cox proportional hazards model and Wald test were used to assess association of GEMM *Kras* signature with outcome variables. Cox proportional hazards model was used also for multivariable model. Survival times were cut at 84 months.

### Gene expression data

All gene expression data can be found at the Gene Expression Omnibus (www.ncbi.nlm.nih.gov/geo/) under accession number GSE50794.

## Supplementary Material

Supplementary Material

## References

[b1-0070613] Arnold C. N. Goel A. Blum H. E. Boland C. R. (2005). Molecular pathogenesis of colorectal cancer: implications for molecular diagnosis. Cancer 104, 2035–2047 16206296 10.1002/cncr.21462

[b2-0070613] Betensky R. A. Louis D. N. Cairncross J. G. (2002). Influence of unrecognized molecular heterogeneity on randomized clinical trials. J. Clin. Oncol. 20, 2495–2499 12011127 10.1200/JCO.2002.06.140

[b3-0070613] Budinska E. Popovici V. Tejpar S. D’Ario G. Lapique N. Sikora K. O. Di Narzo A. F. Yan P. Hodgson J. G. Weinrich S. (2013). Gene expression patterns unveil a new level of molecular heterogeneity in colorectal cancer. J. Pathol. 231, 63–76 23836465 10.1002/path.4212 PMC3840702

[b4-0070613] Cancer Genome Atlas Network (2012). Comprehensive molecular characterization of human colon and rectal cancer. Nature 487, 330–337 22810696 10.1038/nature11252 PMC3401966

[b5-0070613] Clevers H. (2006). Wnt/beta-catenin signaling in development and disease. Cell 127, 469–480 17081971 10.1016/j.cell.2006.10.018

[b6-0070613] Coffee E. M. Faber A. C. Roper J. Sinnamon M. J. Goel G. Keung L. Wang W. V. Vecchione L. de Vriendt V. Weinstein B. J. (2013). Concomitant BRAF and PI3K/mTOR blockade is required for effective treatment of BRAF(V600E) colorectal cancer. Clin. Cancer Res. 19, 2688–2698 23549875 10.1158/1078-0432.CCR-12-2556 PMC3815598

[b7-0070613] de Bono J. S. Ashworth A. (2010). Translating cancer research into targeted therapeutics. Nature 467, 543–549 20882008 10.1038/nature09339

[b8-0070613] De Sousa E. Melo F. Wang X. Jansen M. Fessler E. Trinh A. de Rooij L. P. de Jong J. H. de Boer O. J. van Leersum R. Bijlsma M. F. (2013). Poor-prognosis colon cancer is defined by a molecularly distinct subtype and develops from serrated precursor lesions. Nat. Med. 19, 614–618 23584090 10.1038/nm.3174

[b9-0070613] Dry J. R. Pavey S. Pratilas C. A. Harbron C. Runswick S. Hodgson D. Chresta C. McCormack R. Byrne N. Cockerill M. (2010). Transcriptional pathway signatures predict MEK addiction and response to selumetinib (AZD6244). Cancer Res. 70, 2264–2273 20215513 10.1158/0008-5472.CAN-09-1577 PMC3166660

[b10-0070613] DuPage M. Dooley A. L. Jacks T. (2009). Conditional mouse lung cancer models using adenoviral or lentiviral delivery of Cre recombinase. Nat. Protoc. 4, 1064–1072 19561589 10.1038/nprot.2009.95 PMC2757265

[b11-0070613] Engelman J. A. Chen L. Tan X. Crosby K. Guimaraes A. R. Upadhyay R. Maira M. McNamara K. Perera S. A. Song Y. (2008). Effective use of PI3K and MEK inhibitors to treat mutant Kras G12D and PIK3CA H1047R murine lung cancers. Nat. Med. 14, 1351–1356 19029981 10.1038/nm.1890 PMC2683415

[b12-0070613] Frese K. K. Tuveson D. A. (2007). Maximizing mouse cancer models. Nat. Rev. Cancer 7, 654–658 10.1038/nrc2192 17687385

[b13-0070613] Garnett M. J. Edelman E. J. Heidorn S. J. Greenman C. D. Dastur A. Lau K. W. Greninger P. Thompson I. R. Luo X. Soares J. (2012). Systematic identification of genomic markers of drug sensitivity in cancer cells. Nature 483, 570–575 22460902 10.1038/nature11005 PMC3349233

[b14-0070613] Greenman C. Stephens P. Smith R. Dalgliesh G. L. Hunter C. Bignell G. Davies H. Teague J. Butler A. Stevens C. (2007). Patterns of somatic mutation in human cancer genomes. Nature 446, 153–158 17344846 10.1038/nature05610 PMC2712719

[b15-0070613] Hegde G. V. de la Cruz C. C. Chiu C. Alag N. Schaefer G. Crocker L. Ross S. Goldenberg D. Merchant M. Tien J. (2013). Blocking NRG1 and other ligand-mediated Her4 signaling enhances the magnitude and duration of the chemotherapeutic response of non-small cell lung cancer. Sci. Transl. Med. 5, 171ra118 10.1126/scitranslmed.3004438 23390248

[b16-0070613] Heyer J. Kwong L. N. Lowe S. W. Chin L. (2010). Non-germline genetically engineered mouse models for translational cancer research. Nat. Rev. Cancer 10, 470–480 20574449 10.1038/nrc2877 PMC4602412

[b17-0070613] Hung K. E. Maricevich M. A. Richard L. G. Chen W. Y. Richardson M. P. Kunin A. Bronson R. T. Mahmood U. Kucherlapati R. (2010). Development of a mouse model for sporadic and metastatic colon tumors and its use in assessing drug treatment. Proc. Natl. Acad. Sci. USA 107, 1565–1570 20080688 10.1073/pnas.0908682107 PMC2824379

[b18-0070613] Johnson L. Mercer K. Greenbaum D. Bronson R. T. Crowley D. Tuveson D. A. Jacks T. (2001). Somatic activation of the K-ras oncogene causes early onset lung cancer in mice. Nature 410, 1111–1116 11323676 10.1038/35074129

[b19-0070613] Jonkers J. Berns A. (2002). Conditional mouse models of sporadic cancer. Nat. Rev. Cancer 2, 251–265 12001987 10.1038/nrc777

[b20-0070613] Jorissen R. N. Gibbs P. Christie M. Prakash S. Lipton L. Desai J. Kerr D. Aaltonen L. A. Arango D. Kruhøffer M. (2009). Metastasis-associated gene expression changes predict poor outcomes in patients with Dukes stage B and C colorectal cancer. Clin. Cancer Res. 15, 7642–7651 19996206 10.1158/1078-0432.CCR-09-1431 PMC2920750

[b21-0070613] Kirk R. (2011). Genetics: In colorectal cancer, not all KRAS mutations are created equal. Nat Rev Clin Oncol 8, 1 21218523 10.1038/nrclinonc.2010.204

[b22-0070613] Kirsch D. G. Dinulescu D. M. Miller J. B. Grimm J. Santiago P. M. Young N. P. Nielsen G. P. Quade B. J. Chaber C. J. Schultz C. P. (2007). A spatially and temporally restricted mouse model of soft tissue sarcoma. Nat. Med. 13, 992–997 17676052 10.1038/nm1602

[b23-0070613] Kuraguchi M. Wang X. P. Bronson R. T. Rothenberg R. Ohene-Baah N. Y. Lund J. J. Kucherlapati M. Maas R. L. Kucherlapati R. (2006). Adenomatous polyposis coli (APC) is required for normal development of skin and thymus. PLoS Genet. 2, e146 17002498 10.1371/journal.pgen.0020146 PMC1564426

[b24-0070613] Lee J. T. Gu W. (2010). The multiple levels of regulation by p53 ubiquitination. Cell Death Differ. 17, 86–92 19543236 10.1038/cdd.2009.77 PMC3690487

[b25-0070613] Levine A. J. Puzio-Kuter A. M. (2010). The control of the metabolic switch in cancers by oncogenes and tumor suppressor genes. Science 330, 1340–1344 21127244 10.1126/science.1193494

[b26-0070613] Martin E. S. Belmont P. J. Sinnamon M. J. Richard L. G. Yuan J. Coffee E. M. Roper J. Lee L. Heidari P. Lunt S. Y. (2013). Development of a colon cancer GEMM-derived orthotopic transplant model for drug discovery and validation. Clin. Cancer Res. 19, 2929–2940 23403635 10.1158/1078-0432.CCR-12-2307 PMC3951107

[b27-0070613] Politi K. Pao W. (2011). How genetically engineered mouse tumor models provide insights into human cancers. J. Clin. Oncol. 29, 2273–2281 21263096 10.1200/JCO.2010.30.8304 PMC3731923

[b28-0070613] Pollock C. B. Shirasawa S. Sasazuki T. Kolch W. Dhillon A. S. (2005). Oncogenic K-RAS is required to maintain changes in cytoskeletal organization, adhesion, and motility in colon cancer cells. Cancer Res. 65, 1244–1250 15735008 10.1158/0008-5472.CAN-04-1911

[b29-0070613] Popovici V. Budinská E. Delorenzi M. (2011). Rgtsp: a generalized top scoring pairs package for class prediction. Bioinformatics 27, 1729–1730 21505033 10.1093/bioinformatics/btr233

[b30-0070613] Popovici V. Budinska E. Tejpar S. Weinrich S. Estrella H. Hodgson G. Van Cutsem E. Xie T. Bosman F. T. Roth A. D. (2012). Identification of a poor-prognosis BRAF-mutant-like population of patients with colon cancer. J. Clin. Oncol. 30, 1288–1295 22393095 10.1200/JCO.2011.39.5814

[b31-0070613] Racker E. Resnick R. J. Feldman R. (1985). Glycolysis and methylaminoisobutyrate uptake in rat-1 cells transfected with ras or myc oncogenes. Proc. Natl. Acad. Sci. USA 82, 3535–3538 3858838 10.1073/pnas.82.11.3535 PMC397819

[b32-0070613] Rajalingam K. Schreck R. Rapp U. R. Albert S. (2007). Ras oncogenes and their downstream targets. Biochim. Biophys. Acta 1773, 1177–1195 17428555 10.1016/j.bbamcr.2007.01.012

[b33-0070613] Roper J. Hung K. E. (2012). Priceless GEMMs: genetically engineered mouse models for colorectal cancer drug development. Trends Pharmacol. Sci. 33, 449–455 22739258 10.1016/j.tips.2012.05.001

[b34-0070613] Roth A. D. Tejpar S. Delorenzi M. Yan P. Fiocca R. Klingbiel D. Dietrich D. Biesmans B. Bodoky G. Barone C. (2010). Prognostic role of KRAS and BRAF in stage II and III resected colon cancer: results of the translational study on the PETACC-3, EORTC 40993, SAKK 60-00 trial. J. Clin. Oncol. 28, 466–474 20008640 10.1200/JCO.2009.23.3452

[b35-0070613] Rudrapatna V. A. Cagan R. L. Das T. K. (2012). Drosophila cancer models. Dev. Dyn. 241, 107–118 22038952 10.1002/dvdy.22771 PMC3677821

[b36-0070613] Sadanandam A. Lyssiotis C. A. Homicsko K. Collisson E. A. Gibb W. J. Wullschleger S. Ostos L. C. Lannon W. A. Grotzinger C. Del Rio M. (2013). A colorectal cancer classification system that associates cellular phenotype and responses to therapy. Nat. Med. 19, 619–625 23584089 10.1038/nm.3175 PMC3774607

[b37-0070613] Sumimoto H. Imabayashi F. Iwata T. Kawakami Y. (2006). The BRAF-MAPK signaling pathway is essential for cancer-immune evasion in human melanoma cells. J. Exp. Med. 203, 1651–1656 16801397 10.1084/jem.20051848 PMC2118331

[b38-0070613] Tuveson D. Hanahan D. (2011). Translational medicine: Cancer lessons from mice to humans. Nature 471, 316–317 21412332 10.1038/471316a

[b39-0070613] Tuveson D. A. Jacks T. (2002). Technologically advanced cancer modeling in mice. Curr. Opin. Genet. Dev. 12, 105–110 11790563 10.1016/s0959-437x(01)00272-6

[b40-0070613] Van Cutsem E. Labianca R. Bodoky G. Barone C. Aranda E. Nordlinger B. Topham C. Tabernero J. André T. Sobrero A. F. (2009). Randomized phase III trial comparing biweekly infusional fluorouracil/leucovorin alone or with irinotecan in the adjuvant treatment of stage III colon cancer: PETACC-3. J. Clin. Oncol. 27, 3117–3125 19451425 10.1200/JCO.2008.21.6663

[b41-0070613] Vidal M. Cagan R. L. (2006). Drosophila models for cancer research. Curr. Opin. Genet. Dev. 16, 10–16 16359857 10.1016/j.gde.2005.12.004

[b42-0070613] Yun J. Rago C. Cheong I. Pagliarini R. Angenendt P. Rajagopalan H. Schmidt K. Willson J. K. Markowitz S. Zhou S. (2009). Glucose deprivation contributes to the development of KRAS pathway mutations in tumor cells. Science 325, 1555–1559 19661383 10.1126/science.1174229 PMC2820374

