# Supplementary material for: Cross-species analysis of genetically engineered mouse models of MAPK-driven colorectal cancer identifies hallmarks of the human disease
Source: Dis Model Mech. 2014 Apr 17;7(6):613–23. doi: 10.1242/dmm.013904 (PMC4036469; doi:10.1242/dmm.013904)
Supplement: Supplementary Material [file supp_7_6_613__index.html]

Cross-species analysis of genetically engineered mouse models of MAPK-driven colorectal cancer identifies hallmarks of the human disease — Supplementary Material 

# Cross-species analysis of genetically engineered mouse models of MAPK-driven colorectal cancer identifies hallmarks of the human disease

## DMM013904 Supplementary Material

**Files in this Data Supplement:**

- **Supplementary Material**
