# Supplementary material for: Cross-species analysis of genetically engineered mouse models of MAPK-driven colorectal cancer identifies hallmarks of the human disease
Source: Dis Model Mech. 2014 Apr 17;7(6):613–23. doi: 10.1242/dmm.013904 (PMC4036469; doi:10.1242/dmm.013904)
Supplement: Supplementary Material [file supp_7.6.613_DMM013904.pdf]

Supplemental Figure and Table Legends

Figure S1. Independent validation of GEMM allele specific signatures. GEMM *Apc*, *Tp53*, and *Kras* signatures (A-C, respectively) were applied to an independent GEMM CRC cohort containing overlapping genotypes including A, AK, and AP.

Figure S2. Application of additional GEMM signatures to PETACC-3 dataset. The GEMM *Apc* (A-C) *Tp53* (D-F) and *Braf* (G-I) signatures were applied to the PETACC-3 dataset as described in Figure 5, and OS, RFS, and SAR were compared.

Figure S3. Application of additional GEMM signatures to GSE14333 dataset. The GEMM *Apc* (A-C) *Tp53* (D-F) and *Braf* (G-I) signatures were applied to the GSE14333 dataset as described in Figure 5, and OS, RFS, and SAR were compared.

Table S1. List of allelic combinations represented in primary GEMM tumors analyzed. Description of alleles present in each primary tumor analyzed, as well as numbers of each group and abbreviations.

Tables S2-S3. Enrichment analysis using shared GEMM allele specific multivariable genes from multivariable analysis. Results of MSigDB Analysis using shared multivariable gene lists from the Venn diagram in Figure 2, including shared upregulated *Kras* and *Braf* genes (Table S2) and shared upregulated *Apc* and *Tp53* genes (Table S3).

Tables S4-S7. Enrichment analysis using unique GEMM allele specific multivariable genes from multivariable analysis. Results of MSigDB Analysis using each allele specific multivariable gene list from the Venn diagram in Figure 2, including *Kras* (Table S4), *Braf* (Table S5), *Apc* (Table S6) and *Tp53* (Table S7).

Tables S8-S11. List of top 100 up and downregulated genes which constitute the GEMM allelic gene signatures for *Kras*, *Braf*, *Apc*, and *Tp53*, respectively.

Table S12. Clinical characteristics associated with the GEMM *Kras* signature. The GEMM *Kras* signature was used to determine levels of enrichment within each particular clinical annotation in the PETACC-3dataset.

Table S13. Results of multivariable Cox proportional hazards model studying effect of *Kras* signature (represented by binary variable *Kras*-like), *BRAF* mutation, *KRAS* mutation, grade, mucinous status and MSI on OS, RFS and SAR in stage 3 patient of PETACC-3 trial. The baseline model is non-*KRAS*-like, *BRAF* wt, *KRAS* wt, grade 1-2, non-mucinous and MSS. *Kras* signature remains significant for OS and RFS.

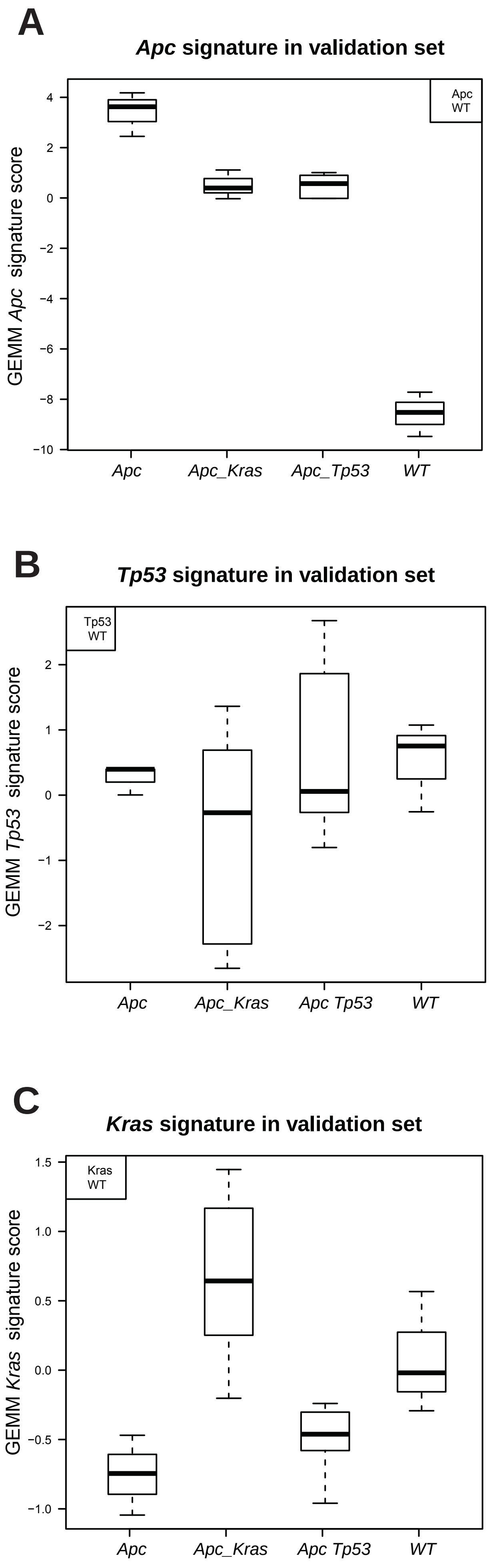
A
Apc signature in validation set
Apc
WT
GEMM Apc signature score
4
2
0
−2
−4
−6
−8
−10
Apc
Apc_Kras
Apc_Tp53
WT
B
Tp53 signature in validation set
Tp53
WT
GEMM Tp53 signature score
2
1
0
−1
−2
Apc
Apc_Kras
Apc Tp53
WT
C
Kras signature in validation set
Kras
WT
GEMM Kras signature score
1.5
1.0
0.5
0.0
−0.5
−1.0
Apc
Apc_Kras
Apc Tp53
WT

Figure S1

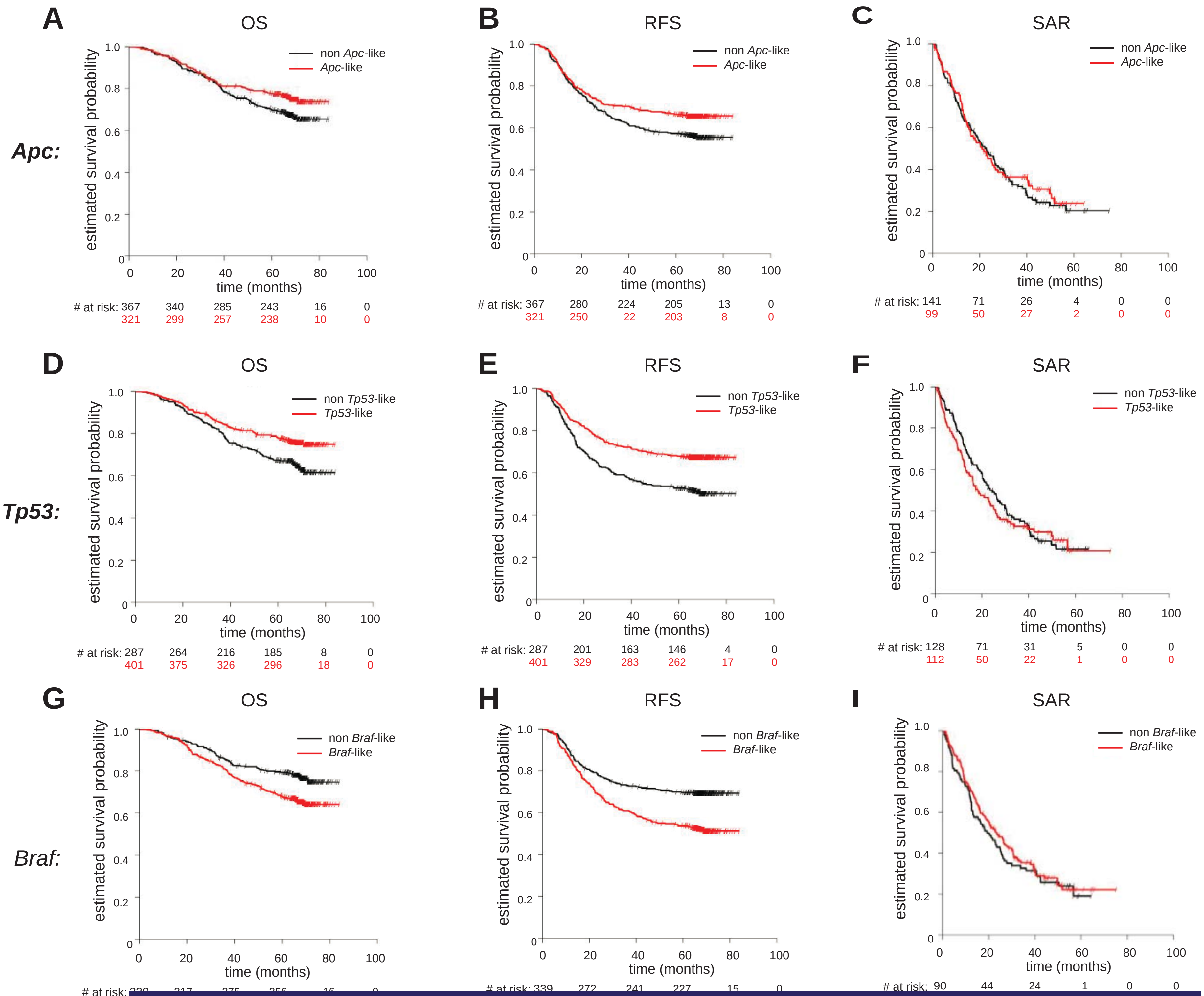

A
OS
Apc:
estimated survival probability
time (months)
non Apc-like
Apc-like
at risk: 367 340 285 243 16 0
321 299 257 238 10 0
B
RFS
non Apc-like
Apc-like
at risk: 367 280 224 205 13 0
321 250 22 203 8 0
C
SAR
non Apc-like
Apc-like
at risk: 141 71 26 4 0 0
99 50 27 2 0 0
D
OS
Tp53:
non Tp53-like
Tp53-like
at risk: 287 264 216 185 8 0
401 375 326 296 18 0
E
RFS
non Tp53-like
Tp53-like
at risk: 287 201 163 146 4 0
401 329 283 262 17 0
F
SAR
non Tp53-like
Tp53-like
at risk: 128 71 31 5 0 0
112 50 22 1 0 0
G
OS
Braf:
non Braf-like
Braf-like
H
RFS
non Braf-like
Braf-like
I
SAR
non Braf-like
Braf-like
at risk: 90 44 24 1 0 0

Figure S2

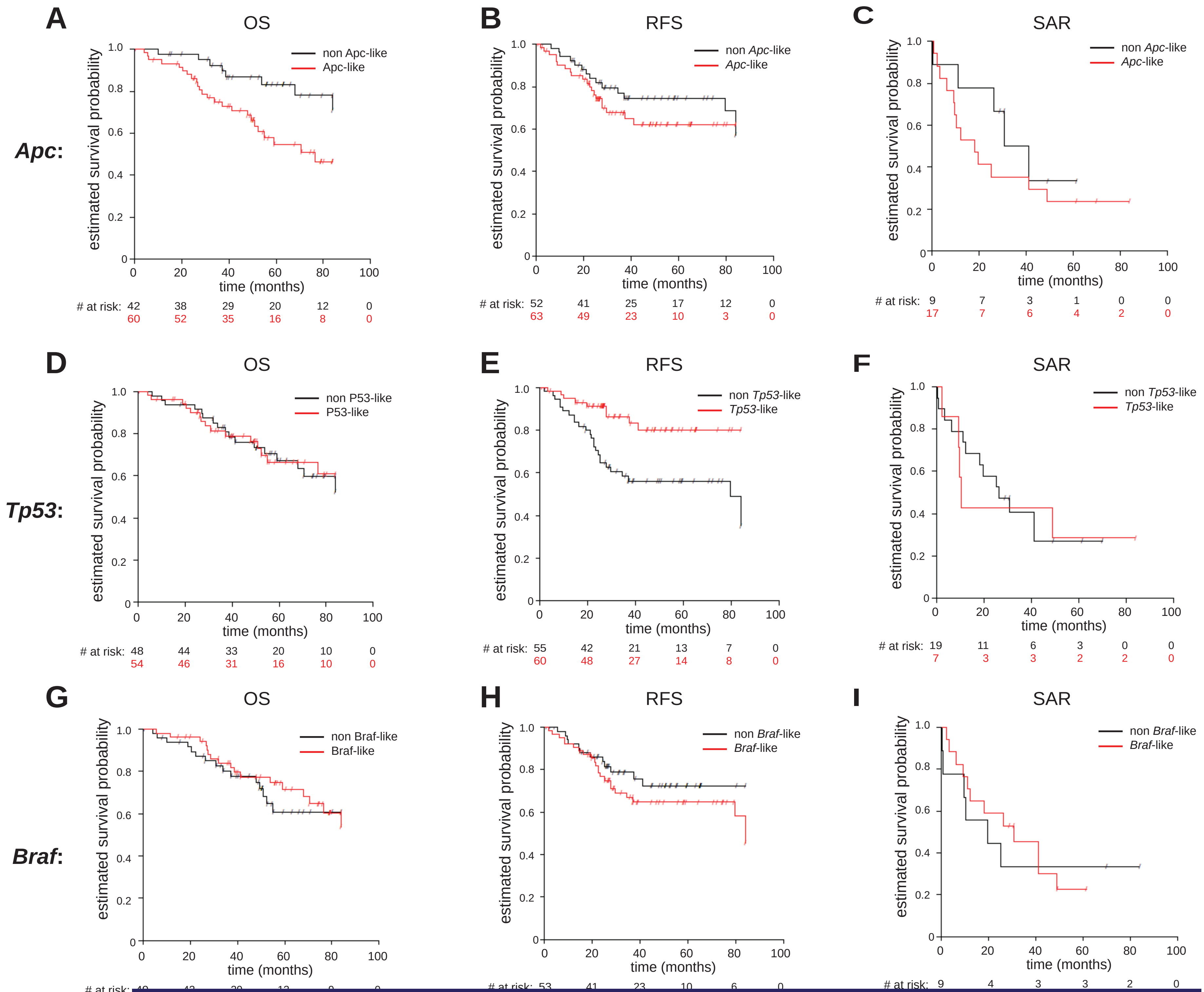
A
OS
Apc:
estimated survival probability
non Apc-like
Apc-like
time (months)
at risk: 42 38 29 20 12 0
60 52 35 16 8 0
B
RFS
non Apc-like
Apc-like
at risk: 52 41 25 17 12 0
63 49 23 10 3 0
C
SAR
non Apc-like
Apc-like
at risk: 9 7 3 1 0 0
17 7 6 4 2 0
D
OS
Tp53:
non P53-like
P53-like
at risk: 48 44 33 20 10 0
54 46 31 16 10 0
E
RFS
non Tp53-like
Tp53-like
at risk: 55 42 21 13 7 0
60 48 27 14 8 0
F
SAR
non Tp53-like
Tp53-like
at risk: 19 11 6 3 0 0
7 3 3 2 2 0
G
OS
Braf:
non Braf-like
Braf-like
at risk:
H
RFS
non Braf-like
Braf-like
at risk: 53 41 23 10 6 0
I
SAR
non Braf-like
Braf-like
at risk: 9 4 3 3 2 0

Figure S3

Download Table S1

Download Table S2

Download Table S3

Download Table S4

Download Table S5

Download Table S6

Download Table S7

Download Table S8

Download Table S9

Download Table S10

Download Table S11

Download Table S12

Download Table S13
